# Mortality Risks and Causes of Death by Dementia Types in a Japanese Cohort with Dementia: NCGG-STORIES

**DOI:** 10.3233/JAD-221290

**Published:** 2023-03-21

**Authors:** Rei Ono, Takashi Sakurai, Taiki Sugimoto, Kazuaki Uchida, Takeshi Nakagawa, Taiji Noguchi, Ayane Komatsu, Hidenori Arai, Tami Saito

**Affiliations:** a Department of Physical Activity Research, National Institutes of Biomedical Innovation, Health and Nutrition, Tokyo, Japan; b Center for Comprehensive Care and Research on Memory Disorders, Hospital, National Center for Geriatrics and Gerontology, Aichi, Japan; c Department of Public Health, Kobe University Graduate School of Health Sciences, Hyogo, Japan; d Department of Prevention and Care Science, Research Institute, National Center for Geriatrics and Gerontology, Aichi, Japan; e Department of Cognition and Behavior Science, Nagoya University Graduate School of Medicine, Aichi, Japan; f Department of Social Science, Research Institute, National Center for Geriatrics and Gerontology, Aichi, Japan; g National Center for Geriatrics and Gerontology, Aichi, Japan

**Keywords:** Alzheimer’s disease, cause of death, frontotemporal lobar degeneration, Lewy bodies, mild cognitive impairment, mortality, prognostic factor, vascular dementia

## Abstract

**Background:**

Prognosis-related information regarding dementia needs to be updated, as changes in medical and long-term care environments for patients with dementia in recent decades may be improving the prognosis of the disease.

**Objective:**

We aimed to investigate the mortality, cause of death, and prognostic factors by types of dementia in a Japanese clinic-based cohort.

**Methods:**

The National Center for Geriatrics and Gerontology-Life Stories of People with Dementia consists of clinical records and prognostic data of patients who visited the Memory Clinic in Japan. Patients who attended the clinic between July 2010 and September 2018, or their close relatives, were asked about death information via a postal survey. A cohort of 3,229 patients (mean age, 76.9; female, 1,953) was classified into six groups: normal cognition (NC), mild cognitive impairment (MCI), Alzheimer’s disease (AD), vascular dementia, dementia with Lewy bodies (DLB), and frontotemporal lobar degeneration. A Cox proportional hazards model was employed to compare the mortality of each type of dementia, MCI, and NC.

**Results:**

Patients with all types of dementia and MCI had higher mortality rates than those with NC (hazard risks: 2.61–5.20). The most common cause of death was pneumonia, followed by cancer. In the MCI, AD, and DLB groups, older age, male sex, and low cognitive function were common prognostic factors but not presence of apolipoprotein E ɛ4 allele.

**Conclusion:**

Our findings suggest important differences in the mortality risk and cause of death among patients with dementia, which will be useful in advanced care planning and policymaking.

## INTRODUCTION

Due to decreasing fertility rates and increasing longevity, the global population is experiencing a structural age shift, especially in developed countries [1]. Of the global burden of serious age-associated health-related conditions, dementia is expected to have the highest proportional increase (264% increase between 2016 and 2060) [2]. Among serious health-related conditions, dementia not only significantly affects individuals but also their families and the entire society. Furthermore, most cases of dementia are incurable and progressive, gradually leading to the loss of cognitive function and ultimately to death. Thus, understanding the prognosis of dementia is important for health policymakers to formulate policies and plans for national resources needed in the management of dementia along with provision of advanced care planning for patients, caregivers, and health professionals.

Previous studies have shown that the median survival time after a diagnosis of dementia varies from 3 to 6 years [3–5], and life expectancy in those with dementia is shorter than that in the general population [6–8]. The dementia type also affects life expectancy. A recent meta-analysis reported that survival time differed between dementia types, with those with dementia with Lewy bodies (DLB) having a shorter survival time than those with Alzheimer’s disease (AD) [9]. Of the dementia types, patients with vascular dementia (VaD) or DLB have been shown to have the shortest survival, followed by patients with AD [7]. Additionally, it has been reported that patients with mild cognitive impairments (MCI) also have shorter survival times than individuals with normal cognition [7, 10, 11]. Prognostic factors of patients with dementia have been reported, including age, sex, number of comorbid conditions, low socioeconomic status, functional disability, and low cognitive function [4, 12–16]. However, few studies have considered prognostic factors or causes of death based on the type of dementia. As mortality rates differ among dementia types, it is necessary to investigate the specific death-related information according to dementia type.

Additionally, previous studies in Asia on prognosis of dementia [17, 18] are limited, and those studies were conducted about a decade ago. The incidence and prevalence trend of dementia varies across countries, decreasing over the last two or three decades in America and Europe [19–21], but increasing in Eastern Asia [17, 22]. However, it is unclear whether this variation is due to differences in race/ethnicity, national systems, and/or maturity levels in countries. As more countries implement dementia-related measures, we have observed progressive improvements in its treatment and diagnosis over the past decade. In Japan, the long-term care insurance system—a welfare system started in 2000 for older people with disability—contributes to supporting people in their late life. Since 2010, all anti-dementia drugs have been available for dementia treatment. Considering changes in these medical and long-term care environments over the last two decades, prognosis of dementia may be improving, and prognosis-related information regarding dementia in Japan needs to be updated.

This study aimed to investigate mortality, cause of death, and prognostic factors according to the type of dementia diagnosis in a Japanese clinic-based cohort (2010–2018). Japan has the highest aging population rate and a well-developed dementia control and care system; therefore, providing evidence from the latest large-scale clinical cohort studies in Japan will have important implications for the trajectory toward dealing with the challenges in dementia.

## METHODS

### Dataset

The National Center for Geriatrics and Gerontology-Life Stories of People with Dementia (NCGG-STORIES) consists of the clinical records and prognostic data of patients who consulted at the Memory Clinic of the NCGG.

This was a retrospective cohort study. The Memory Clinic at the NCGG is one of the six national centers for advanced and specialized medicine, located in one of the most populous prefectures (Aichi) in Japan. There were 4,952 patients in NCGG-STORIES who attended the Memory Clinic at the NCGG between July 2010 and September 2018. We mailed a questionnaire about the patients’ condition to these patients and asked them to complete it and return it via mail from November 2018 to January 2019. Among these, 1,007 did not respond during the study period (response rate: 79.7%). Disagreement or no response to informed consent (*n* = 210) and those who returned the questionnaire but erased their identity number (*n* = 4) were excluded from the study. The identity number was indispensable for the aggregation of clinical and survey data. The study protocol was approved by the local Ethics Committee of the NCGG (No: 1180–2). The purpose, nature, and potential risks of the study were fully explained to the patients or their close relatives, who provided written informed consent before participation.

### Inclusion and exclusion criteria

Participants registered in NCGG-STORIES were included in this study. However, participants were excluded if they had any of the following: missing death information (*n* = 10), idiopathic normal pressure hydrocephalus (*n* = 60), and dementia other than dementia of the unspecified type (*n* = 432).

### Diagnosis of dementia type

Medical doctors diagnosed participants according to dementia type. Participants who had subjective cognitive complaints but were shown to have normal cognitive function on a neuropsychological assessment were diagnosed as having normal cognition (NC). The presence of MCI and dementia was clinically determined according to the criteria of the National Institute on Aging-Alzheimer’s Association workgroups [23, 24]. Dementia was classified as either probable or possible AD [24], probable or possible DLB [25], frontotemporal lobar degeneration (FTLD) [26, 27], VaD [28], or idiopathic normal pressure hydrocephalus [29].

### Information on death

Participants were asked about death information via a postal survey (death-related information, survival status, year and month of death, cause of death, and location of death). The participants or close relatives completed the questionnaires. First, they were asked about the patients’ survival status when they received the questionnaire. If they chose “deceased,” the year, month, and cause of death (cancer, cardiac disease, pneumonia, cerebrovascular disease, others) were requested. We calculated the days until death by assuming that the event occurred on the midday of the month, as we only assessed the year and month of death.

As we obtained information on death in this study from a questionnaire, we verified the accuracy of that information using the patients’ medical records, and a total of 104 patients’ deaths were confirmed. Of these, the survival status of 100 patients were accurately reported (accuracy; 96.2%). The calculated date of death in 93 patients was identical to that in the medical records (accuracy: 93.0%).

### Assessment at consultation

#### Cognitive function

Clinical psychologists assessed cognitive function using the Mini-Mental State Examination (MMSE) [30]. The MMSE scores ranged from 0 to 30, with lower scores indicating impaired cognitive functioning. We operationally divided the scores into three categories (<20, 20–23, and ≥24).

#### Activities of daily living

We assessed activities of daily living (ADL) based on basic and instrumental dimensions. The patients’ caregivers assessed basic ADL (BADL) using the Barthel Index [31]. A higher score indicated greater independence in ADL. Patients with a Barthel Index = 100 were defined as the group with full basic ADL and those with a Barthel Index <100 as the group with basic ADL impairment. Instrumental ADL (IADL) were also assessed by the caregiver using the Lawton Index [32]. The Lawton Index consists of eight items. Each item was scored from “0” (dependent) to “1” (independent). As three of the items of the Lawton Index (“prepare food,” “housecleaning,” and “laundry”) were not applicable to many Japanese men, these three items were excluded when the total score of the Lawton Index was calculated for men. The sum score was divided by five in males and eight in females. Patients with a Lawton Index of 5 and 8 in males and females, respectively, were defined as the group with full IADL, while scores less than these in the respective groups were defined as the group with IADL impairment.

#### Depressive symptoms

Depressive symptoms were assessed by clinical psychologists using the Geriatric Depression Scale 15 [33]. With a score ≥5 indicating mild/severe depressive symptoms, the Geriatric Depression Scale 15 exhibits a sensitivity and specificity of 92.7% and 65.2%, respectively [34].

### Other variables

Demographic variables were collected from the medical records and included age, sex, years of education (≤9 or >9 years), and body mass index (BMI; <18.5, 18.5–<25, or ≥25 kg/m^2^). A portion of the participants had *APOE* phenotypes (*APOE* ɛ4 carrier or *APOE* ɛ4 non-carrier).

### Statistical analysis

Demographic data are expressed for continuous variables as mean and standard deviation and categorical variables as frequencies and proportions. To assess the features of valid respondents, we compared the characteristics between non-analyzed participants and valid respondents using the unpaired *t*-test and χ^2^-test.

We described death as the number of deaths per 1,000 person-years. The Cox proportional hazards model was used to compare each type of dementia, MCI, and NC with the predictor variables—death with age, sex, education, MMSE, BMI, BADL, and IADL—as confounding factors. Hazard ratios and 95% confidence intervals were calculated. Each adjusted hazard ratio was compared using the Bonferroni method (*p* < 0.0033; 0.05/15). To examine the factors related to death by MCI, AD, and DLB, we used the crude and multivariable Cox proportional hazards model to assess the effects of sex, MMSE score, age, education, BMI, BADL, and IADL on death. The multivariable models simultaneously included all variables. We did not apply the Cox models for patients with VaD and FTLD because the sample size was insufficient. To assess the association of *APOE*, we included *APOE* in the Cox proportional hazards model for MCI, AD, and DLB. The Cox proportional hazards model assumption was graphically reviewed using a log-log plot.

Statistical significance was defined as *p* < 0.05. All statistical analyses were performed using STATA version 17.0 (Stata Corp, College Station, TX, USA).

## RESULTS

**Fig. 1:**
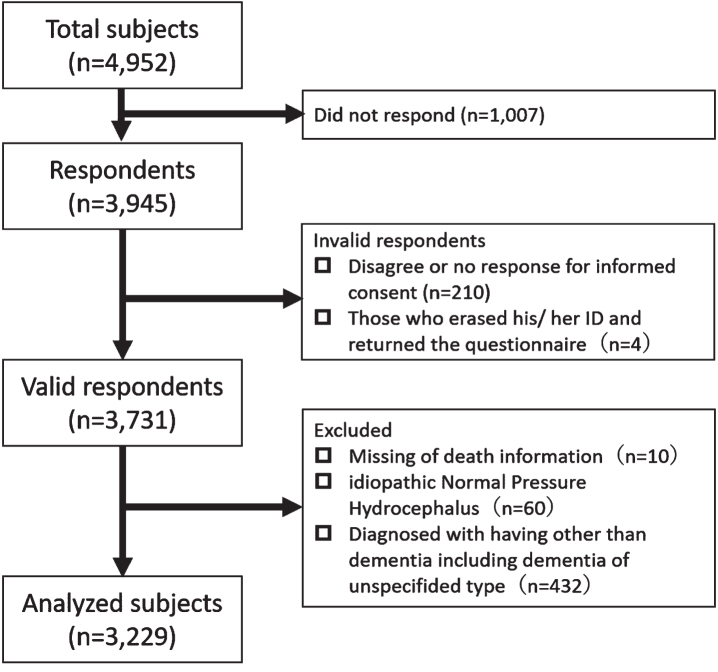
Flow chart in this study.

Finally, 3,731 participants in our cohort were identified as providing valid responses to the questionnaire (Fig. 1). Clinical features of valid respondents compared with non-respondents and invalid respondents are shown in Supplementary Table 1. Valid respondents were more frequently female, lived with others, and had better cognitive function and ADL than did non-respondents and invalid respondents.

Among the valid respondents, 3,229 participants were analyzed (NC = 483, MCI = 865, AD = 1,587, VaD = 76, DLB = 173, and FTLD = 45). Baseline characteristics at diagnosis are shown in Table 1. A total of 564 deaths were recorded (Table 2). The diagnosis with the highest percentage of deaths was DLB (37.6%, 101.80 per 1,000 person-years), followed by VaD (35.5%, 87.28 per 1,000 person-years) and FTLD (31.1%, 85.10 per 1,000 person-years). The causes of death by diagnosis are shown in Table 3. Pneumonia was the leading cause of death in all the diagnoses, MCI and NC, followed by cancer in patients with MCI, AD, and DLB. However, heart disease was the second leading cause of death in patients with VaD. The causes of death that fell under “other causes of death” are shown in Supplementary Table 2.

**Table 1:** Baseline characteristic of patients

Variables		*n*	Total		NC		MCI		AD		VaD		DLB		FTLD	
					(*n* = 483)		(*n* = 865)		(*n* = 1, 587)		(*n* = 76)		(*n* = 173)		(*n* = 45)	
Age, y, mean [SD]		3,229	76.9	[7.9]	70.5	[8.3]	76.0	[7.2]	79.0	[7.1]	78.9	[6.4]	79.6	[6.6]	72.0	[10.0]
Sex, *n* (%)	Female	3,229	1,953	(60.4)	269	(55.7)	469	(54.2)	1,068	(67.3)	25	(32.9)	99	(57.2)	21	(46.7)
	Male		1,278	(39.6)	214	(44.3)	396	(45.8)	519	(32.7)	51	(67.1)	74	(42.8)	24	(53.3)
BMI, kg/m^2^, n (%)	<18.5	3,192	638	(20.0)	38	(7.9)	88	(10.2)	223	(14.1)	6	(7.9)	33	(19.1)	6	(13.6)
	18.5 –<25.0		1,144	(35.8)	353	(73.4)	597	(69.3)	1,084	(68.4)	52	(68.4)	120	(69.3)	30	(68.2)
	≥25.0		1,410	(44.2)	90	(18.7)	177	(20.5)	277	(17.5)	18	(23.7)	20	(11.6)	8	(18.2)
Education, y, n (%)	≤9	3,192	1,411	(44.2)	89	(18.4)	330	(38.5)	845	(54.1)	41	(53.9)	91	(53.5)	15	(34.9)
	>9		1,781	(55.8)	394	(81.6)	528	(61.5)	717	(45.9)	35	(46.1)	79	(46.5)	28	(65.1)
MMSE, *n* (%)	≥24	3,220	1,346	(41.8)	470	(97.3)	591	(68.4)	233	(14.7)	11	(14.5)	30	(17.3)	11	(25.6)
	20–23		612	(19.0)	13	(2.7)	204	(23.6)	345	(21.8)	15	(19.7)	31	(17.9)	4	(9.3)
	<20		1,262	(39.2)	0	(0.0)	69	(8.0)	1,003	(63.4)	50	(65.8)	112	(64.7)	28	(65.1)
GDS, n (%)	<5	3,190	2,012	(63.1)	319	(66.3)	613	(71.1)	951	(60.9)	30	(40.0)	80	(47.1)	19	(47.5)
	≥5		1,178	(36.9)	162	(33.7)	249	(28.9)	611	(39.1)	45	(60.0)	90	(52.9)	21	(52.5)
BADL, n (%)	Full	3,204	2,360	(73.7)	441	(93.6)	750	(87.6)	1,039	(65.5)	23	(30.3)	77	(44.8)	30	(68.2)
	Impaired		844	(26.3)	30	(6.4)	106	(12.4)	546	(34.5)	53	(69.7)	95	(55.2)	14	(31.8)
IADL, n (%)	Full	3,202	1,210	(37.8)	414	(87.7)	523	(61.1)	235	(14.8)	10	(13.2)	19	(11.1)	9	(20.5)
	Impaired		1,992	(62.2)	58	(12.4)	333	(38.9)	1,347	(85.2)	66	(86.8)	153	(88.9)	35	(79.5)
*APOE*, *n* (%)	ɛ4 non-carrier	2,678	1,770	(66.1)	312	(79.8)	463	(68.1)	822	(59.9)	47	(78.3)	105	(71.9)	21	(72.4)
	ɛ4 carrier		908	(33.9)	79	(20.2)	217	(31.9)	550	(40.1)	13	(21.7)	41	(28.1)	8	(27.6)

NC, normal cognition; MCI, mild cognitive impairment; AD, Alzheimer’s disease, VaD, vascular dementia; DLB, dementia with Lewy bodies; FTLD, frontotemporal lobar degeneration; BMI, body mass index; MMSE, Mini-Mental State Examination; GDS, Geriatric Depression Scale; BADL, basic activity of daily living; IADL, instrumental activity of daily living; *APOE*, apolipoprotein E; SD, standard deviation.

**Table 2:** Death-related information by diagnosis

Diagnosis	*n*	Number of deaths	Mean follow-up days	Death per 1,000	Survival time (days)	
		N (%)	(Min, Max)	person years
					25%	50%
NC	483	11 (2.3)	1538.1 (102, 2,989)	5.40	>3000 days	>3000 days
MCI	865	81 (9.4)	1382.4 (57, 3,032)	24.73	>3000 days	>3000 days
AD	1,587	366 (23.1)	1606.9 (50, 2,990)	52.39	2,024	>3000 days
VaD	76	27 (35.5)	1485.7 (78, 2,965)	87.28	1,792	2,397
DLB	173	65 (37.6)	1347.2 (50, 2,925)	101.80	1,296	2,290
FTLD	45	14 (31.1)	1334.0 (210, 2,876)	85.10	1,228	2,586

NC, normal cognition; MCI, mild cognitive impairment; AD, Alzheimer’s disease; VaD, vascular dementia; DLB, dementia with Lewy bodies; FTLD, frontotemporal lobar degeneration.

**Table 3:** Cause of death by diagnosis

Diagnosis	Number of	Cause of death, *n* (%)					
	deaths	Cancer	Heart disease	Pneumonia	Cerebrovascular	Others	Missing
					disease	
NC	11	3 (27.3)	1 (9.1)	3 (27.3)	0 (0)	4 (36.4)	0 (0)
MCI	81	16 (19.8)	10 (12.4)	16 (19.8)	5 (6.2)	33 (40.7)	1 (1.2)
AD	366	68 (18.6)	55 (15.1)	87 (23.8)	31 (8.5)	122 (33.3)	3 (0.8)
VaD	27	4 (14.8)	5 (18.5)	9 (33.3)	2 (7.4)	7 (25.9)	0 (0)
DLB	65	9 (13.9)	3 (4.6)	28 (43.1)	3 (4.6)	19 (29.2)	3 (4.6)
FTLD	14	0 (0)	1 (7.1)	8 (57.1)	1 (7.1)	3 (21.4)	1 (7.1)

NC, normal cognition; MCI, mild cognitive impairment; AD, Alzheimer’s disease; VaD, vascular dementia; DLB, dementia with Lewy bodies; FTLD, frontotemporal lobar degeneration.

In the Cox regression hazard model, using crude and multivariable models, all the types of dementia and MCI were associated with death (adjusted hazard ratios and 95% confidence intervals for MCI: 2.61, 1.37–4.97; AD: 2.70, 1.40–5.22; VaD: 2.84, 1.33–6.05; DLB: 4.57, 2.28–9.14; FTLD: 5.20, 2.21–12.26, respectively) (Table 4). The adjusted hazard ratio of DLB was statistically higher than that of MCI and AD (MCI versus DLB, *p* = 0.0027; AD versus DLB, *p* = 0.0002).

**Table 4:** Relationship between dementia sub-type and mild cognitive impairment, and death using a Cox proportional hazards model

Variables		Crude model		Multivariable model	
		(*n* = 3,229)		(*n* = 3,158)	
		HR	95% CI	HR	95% CI
Dementia sub-type	NC	Ref		Ref
	MCI	4.79	2.55–9.00	2.61	1.37–4.97
	AD	9.40	5.16–17.12	2.70	1.40–5.22
	VaD	15.98	7.92–32.21	2.84	1.33–6.05
	DLB	19.95	10.53–37.80	4.57	2.28–9.14
	FTLD	16.07	7.29–35.39	5.20	2.21–12.26
Age, y				1.07	1.05–1.08
Sex	Female			Ref
	Male			3.18	2.67–3.80
Education, y	≤9			Ref
	>9			1.06	0.89–1.27
MMSE	≥24			Ref
	20–<24			1.37	1.03–1.85
	<20			1.90	1.44–2.50
BMI, kg/m^2^	<18.5			Ref
	18.5–<25.0			0.67	0.53–0.85
	≥25.0			0.52	0.38–0.71
BADL	Full			Ref
	Impaired			1.62	1.35–1.97
IADL	Full			Ref
	Impaired			1.54	1.17–2.02

NC, normal cognition; MCI, mild cognitive impairment; AD, Alzheimer’s disease; VaD, vascular dementia; DLB, dementia with Lewy bodies; FTLD, frontotemporal lobar degeneration; BMI, body mass index; MMSE, Mini-Mental State Examination; GDS, Geriatric Depression Scale; BADL, basic activity of daily living; IADL, instrumental activity of daily living, Multivariate model adjusted for age, sex, education, MMSE, BMI, BADL, and IADL.

To examine death-related factors in MCI, AD, and DLB, we used the Cox proportional hazards model. Regarding the MMSE score, since only a few patients had scores <20 in the MCI group, we re-categorized them from the three classes (<20, 20–23, ≥24) to two classes (<24, ≥24). Since the number of patients in the AD and DLB groups with scores ≥24 was insufficient, we re-categorized them from the three classes (<20, 20–23, ≥24) to two classes (<20, ≥20). In the MCI group, sex, MMSE score, age, and BMI were associated with death. In the AD group, sex, MMSE score, age, education, BMI, and BADL were associated with death. In the DLB group, sex and MMSE scores were associated with death (Table 5).

**Table 5:** Relationship between dementia types and mild cognitive impairment, and death using a Cox proportional hazards model

Variables		MCI				AD				DLB			
		Crude model		Multivariable model		Crude model		Multivariable model		Crude model		Multivariable model	
		(*n* = 864)		(*n* = 851)		(*n* = 1,581)		(*n* = 1,551)		(*n* = 173)		(*n* = 169)	
		HR	95% CI	HR	95% CI	HR	95% CI	HR	95% CI	HR	95% CI	HR	95% CI
Sex	Female	Ref		Ref		Ref		Ref		Ref		Ref
	Male	2.28	1.45–3.59	2.70	1.67–4.37	2.50	2.03–3.06	3.33	2.68–4.12	2.48	1.51–4.06	3.15	1.85–5.37
MMSE	High	Ref		Ref		Ref		Ref		Ref		Ref
	Low	2.00^*^	1.29–3.10	1.71^*^	1.08–2.71	1.87^†^	1.32–2.63	1.47^†^	1.15–1.89	2.19^†^	1.07–4.46	2.41^†^	1.28–4.54
Age, y		1.12	1.08–1.16	1.11	1.07–1.15	1.07	1.06–1.09	1.07	1.05–1.09	1.02	0.98–1.06	1.02	0.98–1.07
Education, y	≤9	Ref		Ref		Ref		Ref		Ref		Ref
	>9	1.19	0.75–1.90	1.30	0.81–2.10	0.90	0.73–1.11	0.63	0.44–0.89	1.14	0.70–1.86	1.25	0.73–2.14
BMI, kg/m^2^	<18.5	Ref		Ref		Ref		Ref		Ref		Ref
	18.5 –<25.0	0.65	0.36–1.18	0.51	0.28–0.95	0.75	0.57–0.99	0.64	0.48–0.85	1.12	0.58–2.15	0.91	0.45–1.81
	≥25.0	0.52	0.24–1.10	0.41	0.19–0.91	0.61	0.42–0.88	0.50	0.34–0.73	0.47	0.15–1.48	0.52	0.16–1.67
BADL	Full	Ref		Ref		Ref		Ref		Ref		Ref
	Impaired	2.75	1.66–4.58	1.64	0.94–2.86	2.23	1.81–2.74	1.72	1.37–2.15	1.87	1.11–3.15	1.25	0.71–2.19
IADL	Full	Ref		Ref		Ref		Ref		Ref		Ref
	Impaired	2.52	1.61–3.94	1.39	0.86–2.25	2.30	1.62–3.31	1.46	0.99–2.15	3.06	0.96–9.75	2.16	0.61–7.60

MCI, mild cognitive impairment; AD, Alzheimer’s disease; DLB, dementia with Lewy bodies; BMI, body mass index; MMSE, Mini-Mental State Examination; GDS, Geriatric Depression Scale; BADL, basic activity of daily living; IADL, instrumental activity of daily living. ^*^MMSE high showed ≥24 and MMSE low showed <24 in MCI. ^†^High MMSE score ≥20 and low MMSE score <20 in AD and DLB. Multivariate models simultaneously included in all variables.

In the Cox proportional hazards model that included *APOE* in the MCI, AD, and DLB groups, being an *APOE* ɛ4 carrier compared with *APOE* ɛ4 non-carrier was not associated with death (adjusted hazard ratios and 95% confidence intervals for MCI: 0.66, 0.39–1.12; AD: 0.82, 0.65–1.03; and DLB: 1.53, 0.88–2.66, respectively (Table 6).

**Table 6:** Relationship between dementia types and mild cognitive impairment, and death using a multivariable Cox proportional hazards model (including *APOE*)

Variables		MCI		AD		DLB	
		(*n* = 669)		(*n* = 1,340)		(*n* = 142)	
		HR	95% CI	HR	95% CI	HR	95% CI
Sex	Female	Ref		Ref		Ref
	Male	2.70	1.66–4.37	3.39	2.72–4.23	2.99	1.73–5.17
MMSE	High	Ref		Ref		Ref
	Low	1.65	1.03–2.63	1.55	1.20–2.00	2.59	1.34–5.02
Age, y		1.10	1.06–1.15	1.06	1.04–1.08	1.03	0.98–1.08
Education, y	≤9	Ref		Ref		Ref
	>9	1.32	0.81–2.13	1.05	0.84–1.31	1.22	0.71–2.11
BMI, kg/m^2^	<18.5	Ref		Ref		Ref
	18.5–<25.0	0.51	0.27–0.94	0.62	0.47–0.83	0.91	0.45–1.82
	≥25.0	0.36	0.16–0.80	0.49	0.33–0.72	0.59	0.18–1.91
BADL	Full	Ref		Ref		Ref
	Impaired	1.55	0.87–2.74	1.69	1.35–2.12	1.34	0.75–2.40
IADL	Full	Ref		Ref		Ref
	Impaired	1.40	0.86–2.28	1.51	1.02–2.24	1.93	0.54–6.89
*APOE*	ɛ4 non-carrier	Ref		Ref		Ref
	ɛ4 carrier	0.66	0.39–1.12	0.82	0.65–1.03	1.53	0.88–2.66

MCI, mild cognitive impairment; AD, Alzheimer’s disease; DLB, dementia with Lewy bodies; BMI, body mass index; MMSE, Mini-Mental State Examination; GDS, Geriatric Depression Scale; BADL, basic activities of daily living; IADL, instrumental activity of daily living; *APOE*, apolipoprotein E. All variables were simultaneously included in each model.

## DISCUSSION

We investigated the prognosis of the types of dementia (AD, VaD, DLB, and FTLD) and MCI by mortality, cause of death, and prognostic factors in a Japanese national center cohort. Compared with NC, all dementia types and MCI were associated with higher mortality rates. The most common causes of death were pneumonia, followed by cancer. In the MCI, AD, and DLB groups, male sex and low cognitive function were common prognostic factors. *APOE* ɛ4 status in patients with dementia subtype and MCI was not associated with death.

Studies have consistently shown that patients with dementia have lower survival rates than those without dementia. The median survival time after a diagnosis of dementia varies largely between studies (from 3.2 to 6.6 years) [3] and differs by dementia subtypes [15, 35]. In the current results, the median survival times for those with AD, VaD, DLB, and FTLD were greater than 3,000, 2,397, 2,290, and 2,586 days, respectively, which was longer than those reported in previous studies [3–5]. Recent reports on AD, VaD, and DLB [4, 7] showed that median survival rates for AD, VaD, and DLB were 5.1–7.4 years, 3.9–4.7 years, and 3.4–4.8 years, respectively. The current results showed a longer median survival time compared to those in previous studies; moreover, the current results are also supported by those of another Japanese study [17] that reported that the 5-year survival rate of all-cause dementia and AD improved from the 1988 to the 2002 cohort. The improvement over time could be due to improvement of the dementia control and care system in Japan.

It has also been reported that the mortality due to MCI and AD has increased compared to that of the general population [7, 18, 36, 37]. Furthermore, DLB also has shorter survival time than AD [9], though the time in VaD and FTLD was shorter than that in AD [7, 38, 39]. In our study, patients with MCI and AD had higher mortality rates than those with NC, the hazard ratio of VaD was similar to that of AD and MCI after adjustment. In the multiple comparisons among the groups, the adjusted hazard ratio of DLB was statistically higher than those of MCI and AD. As the results of this study are similar to those of previous studies, mortality due to the types of dementia may not be affected by race or ethnicity. The following reasons may explain the shorter survival observed in DLB. Patients with DLB have a history of recurrent falls [40] and disease severity, such as more common neuropsychiatric symptoms (including parkinsonism) [40, 41] and/or more frequent delirium [42], and increased mortality. Although the hazard ratio of FTLD was not significantly higher than MCI or AD in this study, studies with more FTLD will be needed.

The present study showed that pneumonia was the main cause of death for patients with all dementia types, and cancer was the second major reason for MCI, AD, and DLB. One study reported that the two most frequent causes of death in a population with dementia (*n* = 207) were pneumonia (34.3%) and acute myocardial infarction (30.4%) [43]. Another study reviewed the effects of pneumonia on the mortality rate of patients with dementia [44]. The risk of pneumonia-associated death in patients with dementia was twice as high as that in those without dementia. A third study found that the most common cause of death was pneumonia (38.4%), followed by ischemic heart disease (23.1%) [45]. From previous studies and the current results, pneumonia has an observably high effect on dementia-related deaths. This may be because in the terminal stages of the disease, patient care and feeding are difficult to manage.

Cancer was the second leading cause of death in the present study. The relationship between cancer and dementia has been reported to be inversely associated with relationship between cancer and AD because of the competing risk [46]. However, these relationships mainly observed the incidence of dementia in patients with diagnosed cancer, and not the incidence of cancer in patients with diagnosed dementia. Additionally, death in patients diagnosed with dementia was not observed. Cancer is a leading cause of death in the Japanese population; as most cancer deaths are among older people (76% and 67% among men and women, respectively) [47]; this may reflect an aged society. Physicians need to pay attention to comorbid cancer in advanced care when planning for dementia in an aged society. Further studies are needed to confirm these results.

In the present study, different prognostic factors affected two types of dementia (AD and DLB) and MCI. Some studies have reported that male sex and older age were prognostic factors [3, 7, 12]. The effect of low cognitive function as measured by the MMSE was not consistently associated with mortality [7, 14]. The current results showed that male sex and low cognitive function were associated with mortality regardless of the type of dementia. Male patients with low cognitive function may require more care or support.

In addition, a low BMI was associated with mortality in patients with MCI and AD. Low nutritional status (malnutrition and underweight) has been reported to be associated with mortality in AD [48]. However, only a few studies have focused on this relationship. Furthermore, the present results can be considered acceptable if a low BMI is considered representative of impaired physical function or sarcopenia.

Moreover, *APOE* ɛ4 carrier status was not associated with mortality in MCI, AD, and DLB. *APOE* ɛ4 carrier status has been shown to lead to a high incidence of AD [49] and DLB [50], but the effect of *APOE* ɛ4 on the risk of dementia and mortality diminishes or disappears with advanced age [51, 52].

This study has several strengths. We were able to investigate various uniform scales for dementia types at baseline and establish a comparison group (NC). As the world’s population ages and the survival rates of patients with dementia along with advances in diagnostic and medical technologies improve, mortality rates and causes of death are expected to change over time. The present results are the latest study findings from Japan, the most aged society in the world.

However, the study also has a few limitations. First, as we collected death-related information via a postal survey, information bias may exist. However, the accuracy of death and the day of death was more than 90%. We believe that the information bias was minimized as much as possible. Second, about one-fourth of the questionnaires were not returned or were invalidated for our survey, suggesting that there may have been a selection bias. Previous studies [53, 54] showed a response rate to mailed epidemiologic questionnaires from 40% to 56%, and the mean response rate in social studies was 67%. Thus, since the response rate of our study exceeds these rates, it is an acceptable rate. Valid respondents were more likely to be female and live with others, exhibit better cognitive function, and have better ADL than non-respondents and invalid respondents. As worsening cognitive function and ADL result in high mortality, our results could be underestimated. As the hazard trends and prognostic factors in this study were similar to those in previous studies, it would indicate the robustness of our results. Third, the cause of death options in our questionnaire was selective. However, the selected cause of death was based on major causes of death in Japan. As the “others” option was freely written by close relatives who might be not well-versed in medical knowledge, that information was not necessarily accurate. Thus, it is necessary to investigate official data, such as death certificates, to determine the cause of death in the future. Fourth, NC group may not be healthy because they sought care at our center with an awareness of cognitive decline. The results compared with NC may be underestimated. Finally, as patients in this study were recruited from a single center and older Japanese patients, the generalizability of their findings would not be guaranteed. Further studies must generalize the results and to capture changes in the progression of dementia care.

In conclusion, in a Japanese national center cohort, we compared mortality rates of patients with various types of dementia and MCI to those of participants with NC. The most common causes of death in patients with dementia and MCI were pneumonia followed by cancer. In the MCI, AD, and DLB, groups, male sex and lower cognitive function were common prognostic factors. *APOE* ɛ4 status in patients with the subtypes of dementia and MCI was not associated with death. Our findings would be useful to caregivers and health professionals as they provide advanced care planning and would also be useful to health policymakers as they make policies and plans for national resources needed in the management of dementia.

## Supplementary Material

Supplementary Material

## Data Availability

The datasets generated during and/or analyzed during the current study are not publicly available due to ethical restrictions, however further analyses may be completed by the authors on reasonable request.
