# Supplementary material for: Mortality Risks and Causes of Death by Dementia Types in a Japanese Cohort with Dementia: NCGG-STORIES
Source: J Alzheimers Dis. 2023 Mar 21;92(2):487–98. doi: 10.3233/JAD-221290 (PMC10041427; doi:10.3233/JAD-221290)
Supplement: Supplementary Material [file jad-92-jad221290-s001.pdf]

Supplementary Material

Mortality Risks and Causes of Death by Dementia Types in a Japanese Cohort with Dementia: NCGG-STORIES

Supplementary Table 1. Patients' characteristics between returners and non-returners

Variables		Number	Valid return (n = 3,731)		Non-return and invalid return (n =1,221)		p
Age, y, mean [SD]		4,949	77.8	[8.1]	76.9	[8.4]	0.4
	Missing		3		0		
Sex, n (%)	Female	4,952	1509	(40.4)	427	(35.0)	<0.001
	Male		2222	(59.6)	794	(65.0)	
Education, y, mean [SD]		4,877	10.9	[2.8]	10.3	[2.6]	<0.001
	Missing		57		18		
BMI, kg/m^2, mean [SD]		4,931	22.2	[3.4]	22.3	[3.5]	0.4
	Missing		15		6		
Number living together, n (%)	0	4,852	412	(11.3)	255	(21.3)	<0.001
	≥1		3243	(88.7)	942	(78.7)	
	Missing		76		24		
Type of dementia, n (%)	NC	4,952	483	(12.9)	91	(7.5)	<0.001
	MCI		867	(23.2)	234	(19.2)	
	AD		1591	(42.6)	593	(48.6)	
	VaD		77	(2.1)	34	(2.8)	
	DLB		174	(4.7)	66	(5.4)	
	FTLD		45	(1.2)	16	(1.3)	
	iNPH		60	(1.6)	14	(1.1)	

	Other		434	(11.6)	173	(14.2)	
MMSE, n (%)	≥ 24	4,936	1534	(41.2)	367	(30.2)	<0.001
	21-23		705	(19.0)	253	(20.8)	
	< 20		1480	(39.8)	597	(49.1)	
	Missing		12		4		
BADL, n (%)	Full	4,898	2626	(71.1)	815	(67.6)	0.024
	Impaired		1067	(28.9)	390	(32.4)	
	Missing		38		16		
IADL, n (%)	Full	4,891	1336	(36.3)	373	(30.9)	<0.001
	Impaired		2348	(63.7)	834	(69.1)	
	Missing		47		14		

NC, normal cognition; MCI, mild cognitive impairment; AD, Alzheimer’s disease; VaD, vascular dementia; DLB, dementia with Lewy bodies; FTLD, frontotemporal lobar degeneration; iNPH, idiopathic normal pressure hydrocephalus; BMI, body mass index; MMSE, Mini-Mental State Examination; GDS, Geriatric Depression Scale; BADL, basic activity of daily living; IADL, instrumental activity of daily living.

Supplementary Table 2. Summary of causes of death included in the “others” option

Specific cause of death
Senility
Accidental death (drowning, traffic accidents)
Asphyxia
Liver cirrhosis
Hepatic encephalopathy
Aorta dissection or rupture
Myelodysplastic syndrome
Sepsis
Pulmonary emphysema
Chronic obstructive pulmonary disease
Heat stroke
Progressive supranuclear palsy
Kidney failure
Multiple organ failure
Fatal arrhythmias
T cell lymphoma
